# Germline Variants in DNA Interstrand-Cross Link Repair Genes May Contribute to Increased Susceptibility for Serrated Polyposis Syndrome

**DOI:** 10.3390/ijms252111848

**Published:** 2024-11-04

**Authors:** Patrícia Silva, Inês Francisco, Bruno Filipe, Pedro Lage, Isadora Rosa, Sofia Fernandes, Ricardo Fonseca, Paula Rodrigues, Joana Parreira, Isabel Claro, Cristina Albuquerque

**Affiliations:** 1 Molecular Pathobiology Research Unit (UIPM), Instituto Português de Oncologia de Lisboa Francisco Gentil E.P.E. (IPOLFG, EPE), Rua Professor Lima Basto, 1099-023 Lisbon, Portugal; palsilva@ipolisboa.min-saude.pt (P.S.); mfrancisco@ipolisboa.min-saude.pt (I.F.); bfilipe@ipolisboa.min-saude.pt (B.F.); 2 Gastroenterology Department, Instituto Português de Oncologia de Lisboa Francisco Gentil E.P.E. (IPOLFG, EPE), Rua Professor Lima Basto, 1099-023 Lisbon, Portugal; plage@ipolisboa.min-saude.pt (P.L.); irosa@ipolisboa.min-saude.pt (I.R.); iclaro@ipolisboa.min-saude.pt (I.C.); 3 Familial Cancer Clinic, Instituto Português de Oncologia de Lisboa Francisco Gentil E.P.E. (IPOLFG, EPE), Rua Professor Lima Basto, 1099-023 Lisbon, Portugal; sffernandes@ipolisboa.min-saude.pt (S.F.); prodrigues@ipolisboa.min-saude.pt (P.R.); mparreira@ipolisboa.min-saude.pt (J.P.); 4 Pathology Department, Instituto Português de Oncologia de Lisboa Francisco Gentil E.P.E. (IPOLFG, EPE), Rua Professor Lima Basto, 1099-023 Lisbon, Portugal; rifonseca@ipolisboa.min-saude.pt

**Keywords:** serrated polyposis syndrome, DNA interstrand-cross link repair

## Abstract

Serrated polyposis syndrome (SPS) is characterized by the development of multiple colorectal serrated polyps and increased predisposition to colorectal cancer (CRC). However, the molecular basis of SPS, especially in cases presenting family history of SPS and/or polyps and/or CRC in first-degree relatives (SPS-FHP/CRC), is still poorly understood. In a previous study, we proposed the existence of two molecular entities amongst SPS-FHP/CRC families, proximal/whole-colon and distal SPS-FHP/CRC, according to the preferential location of lesions and somatic events involved in tumor initiation. In the present study, we aimed to investigate these distinct subgroups of SPS patients in a larger cohort at the germline level and to identify the genetic defects underlying an inherited susceptibility for these two entities. Next-generation sequencing was performed using multigene analysis with a custom-designed panel in a Miseq platform in 60 SPS patients (with and without/unknown FHP/CRC). We found germline pathogenic variants in 6/60 patients (*ATM*, *FANCM*, *MITF*, *RAD50*, *RAD51C*, and *RNF43*). We also found variants of unknown significance (VUS), with prediction of probable damaging effect in 23/60 patients (*ATM*, *BLM*, *BRCA1*, *FAN1*, *ERCC2*, *ERCC3*, *FANCA*, *FANCD2*, *FANCL*, *MSH2*, *MSH6*, *NTHL1*, *PALB2*, *PDGFRA*, *PMS2*, *PTCH1*, *RAD51C*, *RAD51D*, *RECQL4*, *TSC2*, *WRN,* and *XRCC5* genes). Most variants were detected in gene coding for proteins of the Fanconi Anemia (FA) pathway involved in the DNA Interstrand-Cross Link repair (ICLR). Notably, variants in ICLR genes were significantly more frequent in the proximal/whole-colon than in the distal subgroup [15/44 (34%) vs 1/16 (6%), *p* = 0.025], as opposed to the non-ICLR genes that were slightly more frequent in the distal group [8/44 (18%) vs. 5/16 (31%), *p* > 0.05]. Germline defects in the DNA-ICLR genes may contribute to increased serrated colorectal polyps/carcinoma risk in SPS patients, particularly in proximal/whole-colon SPS. The inclusion of DNA-ICLR genes in the genetic diagnosis of SPS patients, mainly in those with proximal/whole-colon lesions, should be considered and validated by other studies. In addition, patients with germline defects in the DNA-ICLR genes may be more sensitive to treatment with platinum-based therapeutics, which can have implications in the clinical management of these patients.

## 1. Introduction

Serrated polyposis (SPS) is a relatively rare condition characterized by the presence of multiple colorectal epithelial lesions with serrated architectures, termed serrated (SE) lesions, and increased predisposition to colorectal cancer (CRC) [1,2,3]. According to the World Health Organization (WHO), the clinical criteria for diagnosing SP, as updated in 2019, are as follows: (i) Presence of at least five serrated lesions/polyps proximal to the rectum, all being at least 5 mm in size, with two or more being at least 10 mm in size; and (ii) >20 serrated lesions/polyps of any size distributed throughout the large bowel, with at least five being proximal to the rectum [1]. SPS is a phenotypically heterogeneous condition, and it has been considered to be a genetic disease; however, the mode of inheritance is not clear, and both recessive and dominant models have been suggested. *MUTYH* germline mutations have been reported in a few SPS cases, although its role in SPS is likely minimal, and other polyposis-related genes like *BMPR1A*, *SMAD4*, *PTEN*, and *GREM1* have also been screened without positive findings [4,5,6]. A germline mutation in *EPHB2* has been suggested as a potential cause in an SPS patient, as well as other candidates like *ATM*, *PIF1*, *RBL1*, *TELO2*, *FBLN2*, *WNK2*, and *XAF1,* identified through whole-exome sequencing and linkage analysis, though these findings remain unconfirmed and unreplicated [7,8,9,10]. These studies uncovered the fact that SPS may occur through unique genetic and molecular mechanisms not previously associated to the serrated pathway of colorectal tumorigenesis, with distinct mutational signatures potentially contributing to tumor formation, although they are sparsely represented [9,11]. Nevertheless, the genetic background underlying SPS is still poorly understood, with only germline mutations in the *RNF43* gene found to be associated to a very small subset of SPS [2,3,12,13,14]. Environmental factors (like smoking, obesity, and diet) and the colonic localization of polyps may be partially responsible for the phenotypic differences and may mold the pattern of inheritance [2,7,14,15,16,17,18]. In a previous study, we performed a comprehensive clinical, histological, and molecular characterization of serrated and adenomatous lesions from a cohort of SPS patients stratified into two groups, with or without/unknown family history of SPS and/or polyps/CRC in first-degree relatives (SPS-FHP/CRC), in order to improve the knowledge regarding familial forms of SPS. We reported that the SPS-FHP/CRC patients present differences with respect to clinical and histological features when compared to the SPS without/unknown FHP/CRC patients. We also proposed that two forms of SPS-FHP/CRC appear to exist, proximal/whole-colon and distal SPS-FHP/CRC, according to the preferential location of the lesions and the somatic events involved in tumor initiation: *MGMT* and mismatch repair (MMR) gene defects, followed by Wnt gene mutations in the former and mutations in the RAS/RAF genes in the latter. This points out the involvement of distinct tumorigenic pathways in these two forms of SP-FHP/CRC and led us to suggest that the early *MGMT* and MMR gene deficiency may underlie an inherited susceptibility to genotoxic stress in the proximal/whole-colon form [19].

Thus, in the present study, we aimed to investigate these distinct subgroups of SPS-FHP/CRC in a larger cohort at the germline level and to identify genes that may be implicated in this condition.

## 2. Results

### 2.1. Clinical Characterization

We found differences regarding the presence of FHP/CRC, namely, the age of diagnosis and the number and type of lesions, and some of these differences had already been described by us previously [19]. The patients with SPS-FHP/CRC had a slightly older average age at diagnosis (i.e., the age at which they presented symptoms) of 58 ± 11 years (range 23–80), while patients with SPS without/unknown FHP/CRC, had an average age at diagnosis of 54 ± 12 years (range 25–73). The number of lesions was higher in the SPS-FHP/CRC group than in the SPS without/unknown FHP/CRC (threshold, ≥40 lesions): [15/30 (50%) vs. 5/28 (18%), *p* = 0.008], as we previously observed in a smaller series [5]. SPS-FHP/CRC tended to have adenomatous lesions more frequently when compared with SPS without/unknown FHP/CRC [27/30 (90%) vs. 22/28 (79%), *p* > 0,05] (Supplementary Table S1).

### 2.2. Germline Variant Characterization

Six pathogenic/likely pathogenic (PV/LPV) variants and 35 VUS with a prediction of probable damaging effect (all heterozygous) were identified in the 60 patients analyzed (Supplementary Table S1). PV/LPV were detected in the *ATM*, *FANCM*, *MITF*, *RAD50*, *RAD51C*, *RNF43* genes. Notably, all PV/LPV were detected in the SPS patients with proximal/whole-colon lesions. Among the VUS, those with probable damaging effects (VUS *) were selected using the in silico tools described in Section 4.4 (Methods). Table 1 summarizes the germline variants, PV/LPV and VUS *, identified in each subgroup of SPS stratified by the presence or absence of FHP/CRC and by the preferential location of the lesions. One patient presented one PV mutation and one VUS with probably damaging effect (A759).

In 16/60 (26%) of the SPS patients, we found variants in genes encoding for proteins of the Fanconi Anemia (FA) pathway (*BLM*, *BRCA1*, *FAN1*, *FANCA*, *FANCD2*, *FANCL*, *PALB2*, *RAD50*, *RAD51C*, *RAD51D*), that are involved in the DNA Interstrand-Cross Link repair (DNA-ICLR). Variants in the DNA-ICLR genes were significantly more frequent in the proximal/whole-colon group than in the distal subgroup [15/44 (34%) vs 1/16 (6%), *p* = 0.025] as opposed to the non-ICLR genes that were slightly more frequent in the distal group [8/44 (18%) vs. 5/16 (31%), *p* > 0.05] (Table 2). The distribution of DNA-ICLR amongst the proximal/whole-colon SPS cases was similar regardless of the presence of FHP/CRC (8/25, 32% vs 7/19, 36%). However, three patients without/unknown FHP/CRC had familial histor-yy of CRC in second-degree relatives, and four patients with DNA-ICLR variants (25%) had a family history of extracolonic tumors, namely, lung, breast, gastric, and liver cancer, mainly in relatives other than first-degree (Supplementary Table S1).

We also found variants in genes coding for DNA nucleotide excision repair (NER), MMR and homologous recombination (HR) genes (*ATM*, *ERCC2*, *ERCC3*, *MSH2*, *MSH6*, *PMS2*, *RECQL4*, *WRN*) in 8/60 (13%). The variants in the NER and MMR genes were found mainly in patients with preferential distal location of lesions and, [4/16, 25% vs 1/44, 2%, *p* = 0.015) in particular, in those patients (3/10, 30%) without/unknown FHP/CRC. Variants in the HR genes were found equally in the proximal/whole-colon and distal SPS cases (3/44, 7% vs 1/16, 6%).

Only one patient had an *RNF43* PV variant (1/60, 2%), and one patient had a VUS with a prediction of probable damaging effect in *NTHL1* (in heterozygosity) (Supplementary Table S1).

Approximately one-third of our cohort (22/60, 37%) had a family history of other tumor types, and the most common extracolonic tumor found in the family history was breast cancer (10/60, 17%) followed by gastric cancer (6/60, 10%), and most of them had a first-degree relative with an extracolonic tumor (15/22, 68%).

## 3. Discussion

In a previous study, we found that SPS-FHP/CRC and SPS without/unknown FHP/CRC had differences at the clinical level, namely, the age of diagnosis, histological pattern, and number of lesions, some of which were also observed in the present study, in particular, the higher number of lesions presented by the SPS-FHP/CRC patients. In the same previous study, we found that a deficient DNA repair pathway characterized by *MGMT* and MMR methylation and/or LOH followed by Wnt gene mutations was predominant at the somatic level, in the proximal/whole-colon SP-FHP/CRC, and we have previously suggested that colonic mucosa with Paneth cell metaplasia may be one of the pre-neoplastic lesions in the development of proximal/whole-colon SPS-FHP/CRC [19]. This molecular somatic signature was further characterized by analyzing the presence of copy-number variations (CNVs) in *MGMT*, *APC,* and six MMR repair genes (*MLH1*, *MSH2*, *MSH6*, *PMS2*, *MLH3*, *MSH3*) by multiplex ligation probe amplification (MLPA) and methylation-specific MLPA (MS-MLPA). We observed a higher frequency of CNVs (mostly gains in the promoter regions of MMR, *MGMT,* and *APC* genes) in serrated and traditional lesions (and even in normal colonic mucosa) from patients with proximal/whole-colon SPS-FHP/CRC (*unpublished data*). These results were compatible with the model that we have proposed for tumor initiation in proximal/whole-colon SP-FHP/CRC, which assumes the existence of futile cycles of replication and repair resulting from an accumulation of errors by oxidative stress/alkylating agents, which is consistent with the fact that proximal colon tumors are characterized by a higher immune response [11,19,20].

In this study, we observed that the majority of patients with proximal/whole-colon SP-FHP/CRC had PV/LPV or VUS with prediction of probable damaging effect (VUS *) in genes coding for proteins of the FA pathway that are involved in DNA-ICLR [21,22,23]. The DNA damage resulting from alkylating agents, when it is not repaired by *MGMT* and MMR genes, may lead to DNA double-strand breaks [23]. These, together with the defects in the DNA-ICLR repair pathway, may result in elevated chromosomal or DNA breakage and genome instability, which is consistent with the previously reported molecular somatic signature of proximal/whole-colon SPS. The difference between the side of the colon appears to matter in CRC: proximal and distal colon differ in their embryonic origin, bile acid metabolism, water resorption, luminal content, bacterial colonization, short-chain fatty acid production, and various other features such as the tumorigenic pathways and immune response [19,20,24]. More recently, differences in the proximal and distal microbiota have also been proposed. Indeed, biofilms appear to be a risk factor for CRC, because their presence on the normal colonic mucosa of patients with sporadic CRC correlated with tumorigenesis at the proximal colon. Bacteria induce interchain cross-link, which increases replication, activating DNA-ICLR and ultimately resulting in DNA double-strand breaks [25]. Also, in this context, inflammation arises, and Paneth cells, known for secreting antimicrobial peptides in response to inflammation, may mark the precursor lesions of intestinal cancer in the context of inflammation and, hence, the precursor lesions in proximal/whole-colon SPS [19,26].

Some studies have already identified germline mutations in some of these genes in in few colorectal cancer patients of large cohorts, most not fully characterized [27,28]; however, in this study, we found that these genes may constitute susceptibility genes within a specific subgroup of patients with SPS and that they seem to correlate with a specific clinical and molecular phenotype/carcinogenic mechanism. Moreover, we have tested some of the first-degree relatives for some of the variants, namely, an affected son of the A187 patient and an affected nephew of CA636, and they presented the variant found in the index patient of their respective families.

Altogether, this led us to propose that germline defects in DNA-ICLR genes may contribute to increase serrated colorectal polyps/carcinoma risk in SPS patients, particularly in proximal/whole-colon SPS. Accordingly, the inclusion of DNA-ICLR genes in the genetic diagnosis of SPS patients, mainly in those with proximal/whole-colon lesions, should be considered and validated by other studies. This can have implications in the clinical management of these patients and respective at-risk relatives in terms of cancer prevention, surveillance, and treatment, as SPS patients with germline defects in their DNA-ICLR genes may be more sensitive to treatment with platinum-based therapeutics.

In addition, we found a *MITF* gene PV in patient A759, a gene associated with melanoma development [29]. However, to our knowledge, the patient has no personal or family history of melanoma. It is not clear if the phenotype presented by this patient may be due to the *MITF* variant, although the same germline mutation found in this patient [p.(Glu425Lys)] was apparently associated to increased risk of developing CRC, especially in second- or third-degree relatives of index patients carrying this germline mutation; however, this was reported in a small number of index patients, and segregation studies were not performed [30].

With respect to germline variants with probable damaging effects in NER/MMR/HR genes, which were also detected in the present study, these may account for a subgroup of SPS patients, mainly in the distal form of SP-FHP/CRC. In fact, germline variants in a few genes of NER and HR pathways have been associated with increased cancer susceptibility, namely, colorectal cancer, although without a well-known associated risk [23,31].

The frequency of the *RNF43* variants observed in this study is in accordance with the described frequency of *RNF43* variants in SPS patients (~2%), representing a small subset of SPS [3].

## 4. Materials and Methods

### 4.1. Study Participants

Sixty patients diagnosed with SPS according to the WHO 2019 diagnostic criteria [1], of which 13 were already characterized molecularly at the somatic level in a previous study [19], were included in this study: 31 index patients with SPS-FHP/CRC (25 with proximal/whole-colon and 6 with a distal preferential location of lesions), and 29 index patients with SPS without/unknown FHP/CRC (19 with proximal/whole-colon and 10 with a distal location of lesions) from the Familial Colorectal Cancer Clinic of the Instituto Português de Oncologia de Lisboa Francisco Gentil. The clinicopathological features of the patients enrolled in this study are described in Supplementary Table S1.

The study was conducted in accordance with local ethical standards and in agreement with the Helsinki Declaration of 1975, as revised in 1983. Informed consent for diagnosis and additional investigational studies, which may result in improving the knowledge about the pathogenesis of the disease, was obtained from the patients included in this study.

### 4.2. DNA Isolation

Genomic DNA was isolated from peripheral blood using the Maxwell^®^ RSC Whole Blood kit (Promega, Madison, WI, USA) in a Maxwell^®^ RSC platform (Promega, Madison, WI, USA) according to the manufacturer’s instructions. The DNA concentration was determined using the Qubit 2.0 fluorimeter (Life Technologies, Waltham, MA, USA).

### 4.3. Germline Variant Analysis

A germline variant analysis was performed using a custom-designed multigene panel, including genes associated with increased cancer risk. The analyzed regions included the coding exons and 20bp of flanking intronic regions on both sides of each exon. Briefly, the genomic DNA samples were enriched for the targeted regions using the hybridization-based protocol described for the Sureselect^XT HS^ Target Enrichment System (Agilent Technologies, Santa Clara, CA, USA). The quality and quantification of the obtained libraries was evaluated using an Agilent Bioanalyzer with a High-Sensitivity DNA Kit (Agilent Technologies, Santa Clara, CA, USA). The libraries were sequenced in a MiSeq platform (Illumina, San Diego, CA, USA) with 75-bp paired-end reads. NGS was performed with a sensitivity of approximately 99% for bases covered to a minimum depth of 20×. Annotation was performed with Surecall v4.2.1.10 (Agilent Technologies, Santa Clara, CA, USA) and analyzed in Alissa Interpret v5.4.2 (Agilent Technologies, Santa Clara, CA, USA) and DECoN v1.0.1 for copy-number variation analysis [32].

### 4.4. Bioinformatics Analysis

Genetic variants were annotated in accordance with the nomenclature of the Human Genome Variation Society (HGVS—http://varnomen.hgvs.org/): the interpretation was performed based on the Single Nucleotide Polymorphism Database (dbSNP), Leiden Open Variation Database (LOVD 3.0), ClinVar database (NCBI), and ACMG classification. Population frequency was verified according to the genome aggregation database (gnomAD). The potential damaging effects of the missense variants to the protein structure and function were predicted in silico by pathogenicity predictor tools (MetaDome, CADD, Eigen, MPA, MaxEntScan, SPiP, dbscSNV, spliceAI, SIFT, Polyphen 2, Fathmm, ClinPred, Mistic, Mutation Taster, LoFtool, DEOGEN2, LIST-S2, LRT, M-CAP, PROVEAN, PON-P2 software), and only variants predicted to be damaging or deleterious in more than 75% of the software were considered as likely damaging. The Integrative Genomics Viewer (IGV) v2.11.2 was used to visualize the variants.

### 4.5. Statistical Analysis

Fisher’s exact test (two-sided) (http://www.quantitativeskills.com/sisa/index.htm, accessed on 17 September 2024) was used to compare the categorical variables. A *p*-value < 0.05 was considered to indicate a statistically significant difference.

## 5. Conclusions

In conclusion, in this study, we identified PV/LPV or VUS with prediction of probable damaging effect in genes involved in the DNA-ICLR pathway that may contribute to increase serrated colorectal polyps or carcinoma risk in a subgroup of familial SPS, namely, those with a preferential location of lesions in the proximal/whole-colon and that present a specific somatic signature characterized by defective *MGMT* and MMR genes. Also, defects in specific NER, MMR, or HR genes may account for a subgroup of SPS patients. It is important to note that the diversity within the study group, when subdivided, results in smaller sample sizes, which may limit the ability to fully capture the range of genetic variation associated with SPS. Therefore, additional studies involving more SPS families are necessary to corroborate the germline genetic susceptibility involvement of these genes in SPS. The validation of these results may have important implications in the clinical management of these patients and their family members, with an obvious impact on cancer prevention. Moreover, this may be relevant to receiving adequate treatment because patients that present defects in DNA-ICLR genes may be more sensitive to treatment with platinum-based therapies.

## Figures and Tables

**Table 1:** Germline variants identified in each subgroup of SPS.

Preferential Location of Lesions	Pathogenicity	Number of Patients with PV/LPV or with VUS *	Germline Variants (PV/LPV or VUS *)
**SPS-FHP/CRC**			
**Proximal/** **whole-colon**	**PV/LPV**	4/25 (16%)	***ATM***: c.4776 + 2T > C ***MITF***: c.1255G > A p.(Glu419Lys) ***RAD51C***: c.890_899del p.(Leu297HisfsTer2) ***RNF43***: c.887dup p.(Asn297Ter)
	VUS *	11/25 (44%)	***BLM***: c.43C > T p.(Arg15Cys); c.2561G > A p.(Ser854Asn) ***BRCA1***: c.4084G > A p.(Asp1362Asn ***FANCA***: c.3551G > C p.(Arg1184Pro) ***FANCD2***: c.2273G > C p.(Cys758Ser) ***MSH2***: c.2210 + 5G > C ***PALB2***: c.101G > A p.(Arg34His) ***PDGFRA***: c.2212G > T p.(Asp738Tyr) ***RAD51D***: c.493C > T p.(Arg165Trp); c.793G > A p.(Gly265Arg) ***RECQL4***: c.1649C > T p.(Ala550Val) ***WRN***: c.272G > C p.(Arg91Thr) ***XRCC5***: c.1123G > T p.(Val375Phe); c.1343-6T > G
**Distal**	VUS *	1/6 (17%)	***PMS2***: c.1004A > G p.(Asn335Ser)
**SPS without/ unknown FHP/CRC**			
**Proximal/** **whole-colon**	**PV/LPV**	2/19 (10%)	***FANCM***: c.2586_2589del p.(Lys863IlefsTer12) ***RAD50***: c.2165dup p.(Glu723GlyfsTer5)
	VUS *	6/19 (32%)	***FAN1***: c.3027dup p.(Gly1010TrpfsTer3) ***FANCA***: c.1874G > C p.(Cys625Ser) ***FANCL***: c.288G > T p.(Lys96Asn) ***PALB2***: c.100C > T p.(Arg34Cys) ***PTCH1***: c.2176C > T p.(Pro726Ser) ***RAD51C***: c.895C > T p.(Pro299Ser)
**Distal**	VUS *	5/10 (50%)	***ATM***: c.2735A > G p.(Gln912Arg) ***ERCC2***: c.1606G > A p.(Val536Met) ***ERCC3***: c.847C > T p.(Arg283Cys) ***FANCA***: c.1038G > C p.(Trp346Cys) ***MSH6***: c.2501G > A p.(Ser834Asn) ***NTHL1***: c.556G > A p.(Ala186Thr) (het) ***TSC2***: c.929A > G p.(Tyr310Cys); c.3971T > C p.(Leu1324Pro)

* VUS with prediction of probable damaging effect.

**Table 2:** Patients with PV/LPV or with VUS* in ICLR and non-ICLR genes.

	Number of Patients with PV/LPV or with VUS * in DNA-ICLR Genes	Number of Patients with PV/LPV or with VUS * in Non-DNA-ICLR Genes
**Proximal/whole-colon**	15/44 (34%) ^1,2^	8/44 (18%) ^2^
**Distal**	1/16 (6%) ^1^	5/16 (31%)

* VUS with prediction of probable damaging effect: ^1^ *p* = 0.025 (Fisher’s exact test) ^2^ *p* = 0.047 (Fisher’s exact test).

## Data Availability

The original contributions presented in this study are included in the article and Supplementary Materials. Further inquiries can be directed to the corresponding author.

## Supplementary Materials

The following supporting information can be downloaded at https://www.mdpi.com/article/10.3390/ijms252111848/s1.
