# Supplementary material for: Germline Variants in DNA Interstrand-Cross Link Repair Genes May Contribute to Increased Susceptibility for Serrated Polyposis Syndrome
Source: Int J Mol Sci. 2024 Nov 4;25(21):11848. doi: 10.3390/ijms252111848 (PMC11546920; doi:10.3390/ijms252111848)
Supplement: Supplementary file 1 [file ijms-25-11848-s001.zip › ijms-3272320-supplementary.pdf]

Supplementary Table S1. Clinicopathological characteristics of SPS patients regarding personal and familial history of polyps and/or CRC and germline genetic variants identified.

Patient ID	Age at diagnosis in years	Gender	Total number of lesions	Type of lesions[a]	Preferential location of lesions[b]	CRC	Family history[d]	Gene	HGVSc	HGVSc
**SPS-FHP/CRC**										
A756	62	M	>100	**HP**+TA+TSA+SSA	Whole-colon†	No	son - SPS;<br>son - polyps	***RAD51C***	c.890_899del	p.(Leu297HisfsTer2)
A755	41	F	>100	**HP**+**TSA**+TVA+SSA+TA	Whole-colon†	No	mother - CRC;<br>maternal aunt - CRC<br>maternal aunt - GIST<br>2 cousins - CRC	-	-	-
CA636	69	M	50	**HP**+TA+TSA+TVA¥	Proximal	Yes*	twin brother - polyps<br>brother - polyps<br>son - polyps;<br>nephew - polyps<br>niece - BC	*BLM*	c.2561G>A	p.(Ser854Asn)
A193	69	F	40	**HP**+TA+TVA	Proximal	Yes	sister - polyps;<br>son - polyp	*FANCD2*	c.2273G>C	p.(Cys758Ser)
A759	50	M	50-100	**HP**+TA	Whole-colon	Yes	mother - polyps<br>brother - polyps<br>maternal uncle - LC	***MITF***<br>*FANCA*	c.1273G>A<br>c.3551G>C	p.(Glu425Lys)<br>p.(Arg1184Pro)
A760	59	F	45	**HP**+**SSA**+TSA	Proximal	No	maternal grandfather - CRC;<br>mother - CRC;<br>sister - polyps<br>daughter - polyps	-	-	-
A1323	59	M	51	**HP+TA**+SSA	Whole-colon	Yes	brother - polyps	*WRN*	c.272G>C	p.(Arg91Thr)
A1612	55	M	100	**HP+TSA**+TA	Whole-colon	Yes	father – CRC<br>paternal cousin – CRC<br>son – polyps<br>paternal grandfather – PcC<br>4 paternal uncles - PC	-	-	-
L1847	49	M	16	**HP**+SSA+TVA<br>prostate cancer (54)	Whole-colon	Yes*	maternal uncle – CRC<br>brother – polyp<br>maternal uncle – PC<br>father – skin<br>paternal grandfather – GC	-	-	-
A2346	71	M	14	**HP+SSA**+TA	Whole-colon	No	mother - CRC;<br>brother - polyps	-	-	-

Supplementary Table S1. (cont)

Patient ID	Age at diagnosis in years	Gender	Total number of lesions	Type of lesions[a]	Preferential location of lesions[b]	CRC	Family history[d]	Gene	HGVSc	HGVSc
A2356	54	M	>20	**HP+SSA**+TA	Whole-colon	No	father – CRC<br>paternal aunt – BC<br>paternal grandfather - GC	-	-	-
A2434	58	F	12	**SSA+HP**	Proximal	No	sister – polyp<br>sister - BC	-	-	-
A3480	78	F	12	**HP+TA**+TSA +TVA+SSA	Proximal	No	brother - CRC	***RNF43***	c.887dup	p.(Asn297Ter)
A3558	69	M	20	**SSA**+TA+TSA+HP	Proximal	Yes	4 CRC<br>1 BC	-	-	-
A3663	51	F	15	**SSA**+TA+HP	Whole-colon	No	brother – polyps<br>sister – polyps<br>mother – OC<br>maternal aunt – OC	-	-	-
A3767	56	M	75	**HP**+TA+TVA+TSA	Whole-colon	No	brother – polyps<br>father – PC<br>mother - TC	*MSH2*<br>*XRCC5*	c.2210+5G>C<br>c.1123G>T	p.(?)<br>p.(Val375Phe)
A3795	61	M	17	**HP**+TA	Proximal	No	father - CRC	-	-	-
A3799	57	M	23	**SSA+HP**+TA	Whole-colon	No	mother - polyps	*BRCA1*	c.4084G>A	p.(Asp1362Asn)
A3891	50	F	30	**HP**+MP+TA+SSA+TVA	Whole-colon	No	father - CRC	*PDGFRA*<br>*RECQL4*	c.2212G>T<br>c.1649C>T	p.(Asp738Tyr)<br>p.(Ala550Val)
A3958	55	M	23	**TSA+HP**+TA	Whole-colon	No	father - CRC	***ATM***	c.4776+2T>C	p.(?)
A4068	23	F	NA	NA	Whole-colon	No	mother – polyp<br>paternal grandfather - CRC	*RAD51D*	c.793G>A	p.(Gly265Arg)
A4170	53	M	55	**HP+TA**+SSA	Whole-colon	No	father – CRC<br>paternal aunt – BC<br>maternal aunt - BC	*PALB2*	c.101G>A	p.(Arg34His)
A4204	46	F	13	**HP**+SSA	Proximal	No	father – CRC<br>maternal uncle - CRC	*BLM*<br>*RAD51D*	c.43C>T<br>c.493C>T	p.(Arg15Cys)<br>p.(Arg165Trp)
A4268	65	M	48	**SSA**+HP+TA+TVA	Proximal	No	SPP	*XRCC5*	c.1343-6T>G	p.(?)
A4302	79	F	>50	**SSA**+AT	Whole-colon	No	paternal grandfather – GC<br>mother – BC<br>sister – CRC<br>brother – RC<br>brother - CNS	-	-	-

Supplementary Table S1. (cont)

Patient ID	Age at diagnosis in years	Gender	Total number of lesions	Type of lesions[a]	Preferential location of lesions[b]	CRC	Family history[d]	Gene	HGVSc	HGVSc
A478	64	M	50-100	**HP**+**TSA**+TA+SSA	Distal[†]	No	father - CRC;<br>sister - polyps;<br>daughter - polyp;<br>son - polyps	-	-	-
A686	80	M	49	**HP**+TSA+TA	Distal	No	2 daughters - polyps;<br>son - polyp	-	-	-
A993	58	F	18	**HP**+TA	Distal	No	mother - CRC;<br>sister - polyps	-	-	-
A983	49	M	45	**HP**+**TSA**+TA+SSA	Distal	No	paternal uncle - polyps;<br>paternal uncle – PC<br>maternal aunt - BC<br>father - polyp;<br>brother - polyp	-	-	-
A2341	51	M	31	**HP**+SSA+TA	Distal	No	Father – CRC<br>Paternal uncle – CRC<br>Paternal aunt - CRC	-	-	-
A4040	56	F	15	**HP+TA**+SSA	Distal	No	Father – CRC, LvC<br>Maternal cousin - CRC	*PMS2*	c.1004A>G	p.(Asn335Ser)
**SPS without FHP/CRC**										
A500	48	F	42	**HP**+**SSA**+TSA	Whole-colon	No	-[‡]	-	-	-
A1213	36	M	21	**HP+SSA+TA**	Proximal	No	-[‡]	-	-	-
A2555	25	M	8	**TSA**+HP+TA	Proximal	No	Maternal grandfather - CRC	*PTCH1*	c.2176C>T	p.(Pro726Ser)
A2696	50	M	41	**HP+TA+SSA**+TVA	Whole-colon	No	Father – LvC<br>Paternal cousin - GC	*PALB2*	c.100C>T	p.(Arg34Cys)
A2824	50	F	22	**SSA+HP**+TA	Proximal	No	Paternal grandfather - CRC	*RAD51C*	c.895C>T	p.(Pro299Ser)
A3160	62	M	30-50	**HP+TA+TVA**	Whole-colon	Yes	-[‡]	-	-	-
A3470	41	M	21	**TSA**+HP+TA	Whole-colon	No	-[‡]	-	-	-
A3475	55	F	33	**HP+SSA**+TA	Whole-colon	No	Maternal grandfather - CRC	*FANCL*	c.288G>T	p.(Lys96Asn)
A3494	69	M	>21	**HP+SSA**+TA	Whole-colon	No	-[‡]	*FANCA*	c.1874G>C	p.(Cys625Ser)
A3662	52	M	19	**HP+SSA**+TA	Whole-colon	No	Father - CNS	-	-	-
A3791	58	F	16	**HP+SSA**+TA	Whole-colon	No	Mother – BC<br>Maternal cousin – BC<br>Maternal aunt - OC	-	-	-

Supplementary Table S1. (cont)

Patient ID	Age at diagnosis in years	Gender	Total number of lesions	Type of lesions[a]	Preferential location of lesions[b]	CRC	Family history[d]	Gene	HGVSc	HGVSc
A3803	57	M	26	**HP+TA**	Whole-colon	No	Mother - PcC	-	-	-
A3804	68	M	NA	**TSA**+TA	Proximal	Yes*	-[‡]	-	-	-
A3867	47	F	24	**SSA+TA**+TVA+HP	Whole-colon	No	Maternal grandmother - CRC	***RAD50***	c.2165dup	p.(Glu723GlyfsTer5)
A3949	56	F	13	**SSA+HP**+TVA	Whole-colon	No	-[‡]	-	-	-
A4110	54	F	14	**HP**+TA+SSA	Whole-colon	No	-[‡]	*FAN1*	c.3027dup	p.(Gly1010TrpfsTer3)
A4117	73	M	11	**SSA**+TA+TVA	Whole-colon	No	Mother – RN	-	-	-
A4131	68	M	9	**SSA**+HP	Whole-colon	No	-[‡]	-	-	-
A5060	40	M	10	**SSA**+TA+TVA	Whole-colon	No	-[‡]	***FANCM***	c.2586-2589del	p.(Lys863IlefsTer12)
A757	53	M	50	**HP+TSA**	Distal	Yes*	Sister - BC	*ERCC2*	c.1606G>A	p.(Val536Met)
A992	46	F	10	**TSA+HP**	Distal	No	Mother - meningioma<br>Maternal aunt - LvC<br>2 Maternal uncles – GC	*ERCC3*<br>*ATM*<br>*TSC2*<br>*TSC2*	c.847C>T<br>c.2735A>G<br>c.929A>G<br>c.3971T>C	p.(Arg283Cys)<br>p.(Gln912Arg)<br>p.(Tyr310Cys)<br>p.(Leu1324Pro)
A3552	57	F	18	**HP**+TA+TSA+SSA	Distal	No	Maternal uncle – CRC<br>Mother - GC	-	-	-
A3555	72	M	>24	**SSA**+TA	Distal	Yes	-[‡]	-	-	-
A3692	56	M	>20	**HP**	Distal	No	Mother - GC	-	-	-
A3844	34	M	6	**SSA**	Distal	No	-[‡]	-	-	-
A3895	52	M	39	**HP**	Distal	No	-[‡]	*NTHL1*	c.556G>A	p.(Ala186Thr) (het)
A4019	73	M	60	**HP**+TA+TVA	Distal	No	Sister - BC	-	-	-
A4020	73	M	8	**SSA+PH**+TA+TVA	Distal	No	Cousin – NHL	*MSH6*	c.2501G>A	p.(Ser834Asn)
A4187	45	M	23	**HP+SSA**+TA	Distal	No	-[‡]	*FANCA*	c.1038G>C	p.(Trp346Cys)

CRC, colorectal cancer; M, male; F, female; HP, hyperplastic polyp; TA, tubular adenoma; TSA, traditional serrated adenoma; SSA, sessile serrated adenoma; [†],The polyps are larger in the proximal colon; SE, serrated; TVA, tubulovillous adenoma; TSA, traditional serrated adenoma; VA, villous adenoma; *, AD/S, adenomatous/serrated carcinoma; AD, adenomatous; [¥], with areas of TSA; [‡], No evidence of SPS and/or polyps/CRC was found in first degree relatives of the sporadic SPS patients, either by regular colonoscopy examination or by absence of symptoms; NA, not available; MP, mixed polyp; BC, breast cancer; LC, lung cancer; GIST, gastrointestinal stromal tumor; PcC, pancreatic cancer; PC, prostate cancer; GC, gastric cancer; OC, ovarian cancer; TC, thyroid cancer; RC, renal cancer, central nervous system cancer; LvC, liver cancer, NHL, non-Hodgkin lymphoma

[a]The type of lesion(s) that was prevalent (≥70%) in each patient is indicated in bold.

[b]We considered a proximal or distal preferential location of the lesions when at least 70% of the lesions (majority serrated) were located in the proximal or distal colon, respectively.
